# Folate Receptor Beta as a Direct and Indirect Target for Antibody-Based Cancer Immunotherapy

**DOI:** 10.3390/ijms22115572

**Published:** 2021-05-25

**Authors:** Allison G. Roy, J. Michael Robinson, Prannda Sharma, Alba Rodriguez-Garcia, Mathilde A. Poussin, Cheryl Nickerson-Nutter, Daniel J. Powell

**Affiliations:** 1 Ovarian Cancer Research Center, Department of Pathology and Laboratory Medicine, Perelman School of Medicine, University of Pennsylvania, Philadelphia, PA 19104, USA; smith20a@gmail.com (A.G.R.); pranndas@pennmedicine.upenn.edu (P.S.); rodriguez6@clinic.cat (A.R.-G.); mpoussin@pennmedicine.upenn.edu (M.A.P.); 2 Center for Cellular Immunotherapies, Abramson Cancer Center, University of Pennsylvania, Philadelphia, PA 19104, USA; 3 Department of Gynecologic Oncology, Zimmer Cancer Center, New Hanover Regional Medical Center, Wilmington, NC 28401, USA; mike.johnmr@gmail.com; 4 Three Lakes Foundation, Northbrook, IL 60062, USA; CNutter@threelakespartners.org

**Keywords:** folate receptor beta, acute myeloid leukemia, ovarian cancer, tumor-associated macrophages

## Abstract

Folate receptor beta (FRβ) is a folate binding receptor expressed on myeloid lineage hematopoietic cells. FRβ is commonly expressed at high levels on malignant blasts in patients with acute myeloid leukemia (AML), as well as on M2 polarized tumor-associated macrophages (TAMs) in the tumor microenvironment of many solid tumors. Therefore, FRβ is a potential target for both direct and indirect cancer therapy. We demonstrate that FRβ is expressed in both AML cell lines and patient-derived AML samples and that a high-affinity monoclonal antibody against FRβ (m909) has the ability to cause dose- and expression-dependent ADCC against these cells in vitro. Importantly, we find that administration of m909 has a significant impact on tumor growth in a humanized mouse model of AML. Surprisingly, m909 functions in vivo with and without the infusion of human NK cells as mediators of ADCC, suggesting potential involvement of mouse macrophages as effector cells. We also found that TAMs from primary ovarian ascites samples expressed appreciable levels of FRβ and that m909 has the ability to cause ADCC in these samples. These results indicate that the targeting of FRβ using m909 has the potential to limit the outgrowth of AML in vitro and in vivo. Additionally, m909 causes cytotoxicity to TAMs in the tumor microenvironment of ovarian cancer warranting further investigation of m909 and its derivatives as therapeutic agents in patients with FRβ-expressing cancers.

## 1. Introduction

Traditionally, cancer treatment has involved various combinations of surgery, radiation, and chemotherapy. However, the emergence of immunotherapies and targeted therapies, particularly in the past two decades, has transformed the treatment of many different types of cancer. Immunotherapy for cancer treatment can be implemented in a variety of different mechanisms including the use of direct antibodies against various immunomodulators or cancer antigens, adoptive therapies like tumor-infiltrating lymphocytes or chimeric antigen receptor (CAR) T cells, as well as cancer vaccines [1]. As immunotherapy research has expanded, the role of the tumor microenvironment in the proliferation and invasiveness of tumors has also become of increasing interest, particularly, the role of tumor-associated macrophages (TAMs).

A promising target for immunotherapy is FRβ. FRβ is a member of the folate receptor family, which includes four different folate binding receptors (α, β, γ, and δ). Folate is essential for the biosynthesis of nucleotide bases and for many methylation reactions. Both folate receptor alpha (FRα) and folate receptor beta (FRβ) have been shown to be upregulated in rapidly dividing cells such as those associated with malignancy [2,3,4]. FRα has been a well-studied target of immunotherapies for over two decades and continues to be optimized with FRα targeted CAR T cells [5,6], an FRα-specific antibody (Farletuzumab) [7,8,9,10,11,12], and FRα-specific drug conjugated antibodies (mirvetuximab soravtansine) [13,14,15] all being evaluated in clinical trials. FRβ and FRα are both glycosyl phosphatidylinositol (GPI)-bound, share ~70% homology, have a similar affinity for folate, and have a common mechanism of receptor endocytosis-mediated folate uptake [3,4]. While FRα is primarily expressed on epithelial tissues, making it a target in some solid tumors, FRβ is expressed on myeloid lineage hematopoietic cells [16] and has been shown to be expressed in up to 70% of cases of acute myeloid leukemia [17] making it a potential therapeutic target.

One of the important myeloid lineage cells that FRβ is expressed on are TAMs. TAMs can be polarized to an M1 or proinflammatory/anti-tumor subtype or an M2 or immunosuppressive/pro-tumor subtype [18]. TAMs can potentially differentiate into either of these subtypes but the soluble chemokines and cytokines (CCL2, MCSF, IL4, IL10, and TGFB) in the tumor microenvironment favor the M2 polarization [19]. M2 macrophages secrete growth factors, matrix metalloproteases, pro-angiogenic factors, and express inhibitory and immunosuppressive cytokines resulting in tumor growth, angiogenesis, metastasis, and evasion of immune recognition [19]. The presence of TAMs and particularly the increased M2 polarization is associated with a poorer prognosis in many types of cancer including ovarian cancer [20,21,22]. The presence of M2 TAMs in ovarian cancer is correlated with higher stage [23], higher grade [23,24], and shorter survival [25]. FRβ has been shown to be a specific marker for M2 polarized macrophages in the tumor microenvironment [26].

m909 is an FRβ-specific human monoclonal antibody that was shown to recognize activated macrophages from rheumatoid patients and to mediate antibody-dependent cell-mediated cytotoxicity (ADCC) [27]. However, its role in oncology is less clear. Using sequences from m909, we previously created an FRβ targeted CAR T cell approach that demonstrated activity in both in vitro and in vivo models of AML without evidence of hematopoietic stem cell toxicity [28,29]. Based on the effectiveness of these FRβ CAR T cells in the preclinical setting and the improved ease of production and administration of direct antibody therapies compared to CAR T cells, we hypothesized that the parental anti-FRβ antibody (m909) could be a potential treatment for AML by recognizing FRβ on AML blast cells and mediating the toxicity of these cells. Additionally, an antibody against FRβ could be used to improve the treatment of solid tumors such as ovarian cancer by eliminating immunosuppressive, pro-tumorigenic M2 macrophages in the microenvironment. Therefore, we sought to demonstrate the potential of targeting FRβ with m909 as both direct treatment of AML and indirect treatment of ovarian cancer.

## 2. Results

### 2.1. m909 Detects FRβ Expression and Can Mediate ADCC of AML Cell Lines

**Figure 1:**
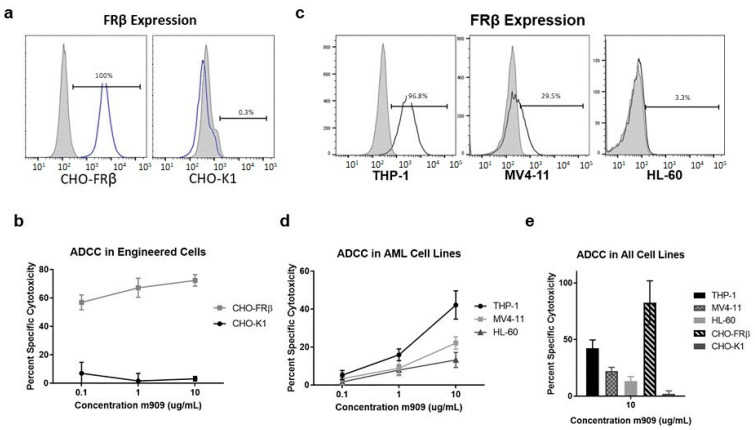
FRβ expression analysis and targeting using m909 antibody. (**a**) FRβ is expressed at 100% on the engineered cell line (CHO-FRβ). No expression is detected in the parental control (CHO-K1). (**b**) Antibody-dependent cytotoxicity assay (ADCC) with CHO-FRβ cells, NK cells at E:T ratio 10:1 and increasing doses of m909. Specific cytotoxicity is calculated by subtracting the percentage of cell death with no antibody. Representative results from one of two independent experiments with triplicate samples in each experiment are shown. (**c**) FRβ expression on immortalized AML cell lines THP-1, MV4-11, and HL-60. (**d**) ADCC assay at increasing doses of m909 for AML cell lines incubated with NK cells at E:T of 10:1 demonstrating a dose-dependent and expression-dependent response. Representative results from one of three independent experiments with triplicate samples per concentration and cell type are shown. (**e**) Specific cytotoxicity for ADCC assay for each cell line at 10 μg/mL m909 using NK cells from the same donor performed with triplicate samples. All error bars represent standard deviation.

FRβ surface expression was evaluated on engineered cell lines and AML cell lines. m909 was biotinylated to allow for detection of FRβ surface expression on the various cell lines via flow cytometry. The expression of FRβ was first analyzed on engineered and parental CHO cell lines. The parental CHO-K1 cells, used as a control, did not express detectable FRβ while all CHO-FRβ cells, had detectable surface FRβ expression (Figure 1a). To validate the ability of m909 to cause FRβ-targeted ADCC, CHO-FRβ and CHO-K1 cells expressing green fluorescent protein and luciferase (GFP-Luc) were co-cultured with NK cells in the presence of m909. Cytotoxicity was determined by residual luciferase expression after approximately 24 h. ADCC studies performed at 0.1 μg/mL, 1 μg/mL, and 10 μg/mL antibody concentrations showed significant cytotoxicity of CHO-FRβ cells even at the lowest concentration of antibody. A 56.9% specific cytotoxicity at 0.1 μg/mL and 72.4% specific cytotoxicity at 10 μg/mL of m909 was observed. There was no evidence of substantial cytotoxicity in CHO-K1 cells after m909 treatment at any antibody concentration (Figure 1b).

The expression of FRβ and the induction of ADCC by m909 in immortalized human AML cell lines were then evaluated to understand the impact of m909 on cells with natural expression of FRβ. The human AML cell line THP-1 expressed high levels of FRβ while MV4-11 cells expressed a moderate level, and HL-60 cells had minimal to no detectable expression of FRβ (Figure 1c). These cell lines were transduced for GFP-Luc expression and ADCC assays were performed in a similar fashion to that for the CHO-K1 and CHO-FRβ cells. THP-1 cells were sensitive to m909-mediated cytotoxicity with 42.2% cell killing observed at the highest antibody concentration while 22.1% specific cytotoxicity was achieved against MV4-11 cells, and 13.2% against HL-60 cells. All cell lines demonstrated a dose-dependent response. (Figure 1d). Using NK cells from the same donor, the increase in specific cytotoxicity for all cell lines also correlated with increasing surface expression of FRβ (Figure 1e).

### 2.2. m909-Mediated Cytotoxicity of AML Cells Relies upon ADCC

**Figure 2:**
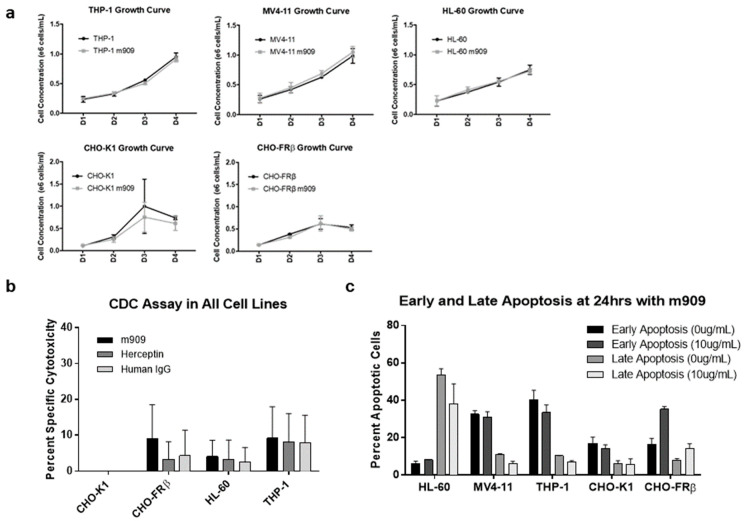
Evaluating additional mechanisms of action for m909 antibody. (**a**) Cells grown in the presence or absence of 10 μg/mL of m909 over time in days. (**b**) Complement-dependent specific cytotoxicity for cell lines in presence of 10 μg/mL of m909, Herceptin, or human IgG1 with human serum. Samples tested in triplicate. (**c**) Percentage of cells with early and late markers of apoptosis after 24 h of incubation with and without 10 μg/mL m909. All error bars represent standard deviation.

Having determined that m909 can effectively induce FRβ-targeted cell death through ADCC, we investigated whether m909 could mediate cell death by alternative mechanisms. We first assessed whether the presence of the antibody in the absence of effector cells affected the growth of FRβ-expressing cells in culture. CHO-FRβ, CHO-K1, THP-1, MV4-11, and HL-60 cells were cultured in the presence or absence of m909 and growth curves were plotted. No significant differences were observed in overall cell growth in the presence or absence of m909 (Figure 2a).

Next, we examined the effect of m909-directed complement-dependent cytotoxicity (CDC) on FRβ high and low expressing cell lines transduced with GFP-fLuc. In addition to m909, cells were treated with non-specific antibodies, Herceptin and Human IgG1, as controls. m909 did not mediate increased CDC against the various cell targets compared to control antibodies, as determined by a paired t-test (Figure 2b). The lack of cytotoxicity observed in the CDC experiments indicates that m909 appears to function independently of the need for complement activation.

Finally, we evaluated the potential for m909 to cause direct cell apoptosis, albeit this seemed an unlikely method of action for m909 based upon cell growth results. After culturing the various cell lines in the presence or absence of 10 μg/mL of m909 for a 24-h incubation, the percentage of cells in early apoptosis and those in late apoptosis were determined and compared. For some cell lines, greater than 50% of the cells showed signs of apoptosis, however, there were no significant differences between cells treated with m909 and the untreated cells across all the cell lines (Figure 2c), indicating that m909 binding to FRβ on the cancer cell surface does not mediate direct cell apoptosis in vitro.

### 2.3. AML Patient Samples Express FRβ and m909 Can Cause ADCC of These Samples

**Figure 3:**
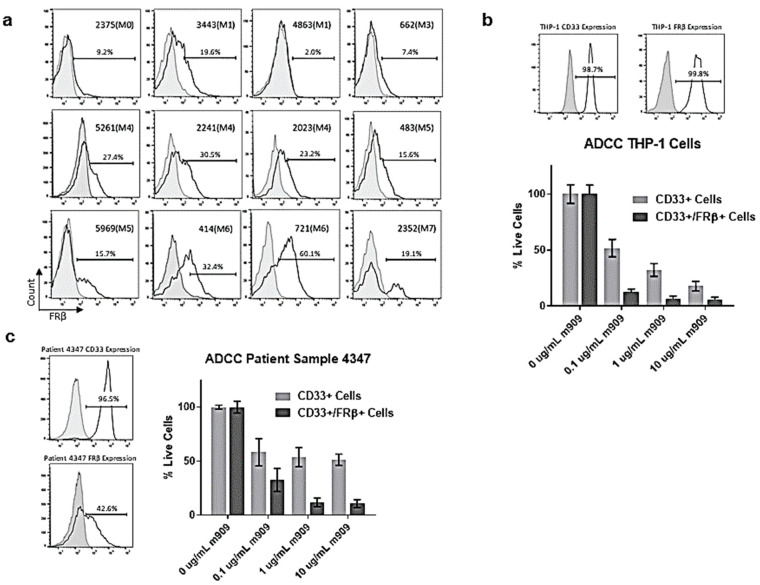
Activity of m909 against primary patient AML samples. (**a**) FRβ expression on patient samples with various subtypes of AML. (**b**) Expression of CD33 and FRβ in THP-1 cells and ADCC by flow using THP-1 cells demonstrates efficacy with dose-dependent response. (**c**) CD33 and FRβ expression data for patient sample 4347. ADCC data for patient sample 4347 showing average from two separate experiments with triplicate samples. All graphs were normalized to wells with 0 μg/mL of m909. Error bars represent standard deviation.

In order to assess the applicability of m909 to AML patients rather than just immortalized cell lines, we also studied the expression of FRβ in patient samples with various subtypes of AML. From previous expression studies in our lab and by others [3,17,30], it has been demonstrated that M4 and M5 subtypes of AML tend to express the highest levels of FRβ. However, in this study, the majority of samples expressed at least moderate levels of FRβ with an average of 21.9% expression and a range of 2.0–60.1% among the 12 samples (Figure 3a). Due to the inability to transduce these cells with GFP-Luc to perform ADCC in the same manner as the immortalized cell lines, we developed a method using flow cytometry to detect the number of viable cells remaining. We first validated a flow-based assay using THP-1 cells as targets. THP-1 cells were plated in the presence of the indicated concentrations of m909 and co-cultured overnight with NK cells. Subsequent staining with fluorophore-labeled anti-CD33 was used to identify the THP-1 cells and biotinylated m909 was used to detect FRβ. The cells were then analyzed using flow cytometry for the number of live CD33+ and CD33+/FRβ+ cells at each concentration. Treatment resulted in a reduction in CD33+ cells of 48.1% at 0.1 μg/mL, 67.8% at 1 μg/mL, and 82.2% at 10 μg/mL of m909. When staining for CD33+/FRβ+ cells, the decrease was even greater with 87.6% at 0.1 μg/mL, 93.4% at 1 μg/mL, and 94.2% at 10 μg/mL (Figure 3b).

Next, a patient sample with high levels of CD33 expression and moderate levels of FRβ expression was selected for this method of analysis. Following treatment with m909, the patient sample showed a statistically significant reduction of the amount of live CD33+ cells of 41.7% at 0.1 μg/mL, 46.0% at 1 μg/mL, and 48.6% at 10 μg/mL, compared to the untreated control. The reduction in CD33+/FRβ+ cells was even greater with 67.2% at 0.1 μg/mL, 88.1% at 1 μg/mL, and 89.2% at 10 μg/mL, demonstrating a concentration-dependent effect on cytotoxicity mediated by m909 (Figure 3c).

### 2.4. m909 Reduces Tumor Growth in a Human AML Xenograft Model

**Figure 4:**
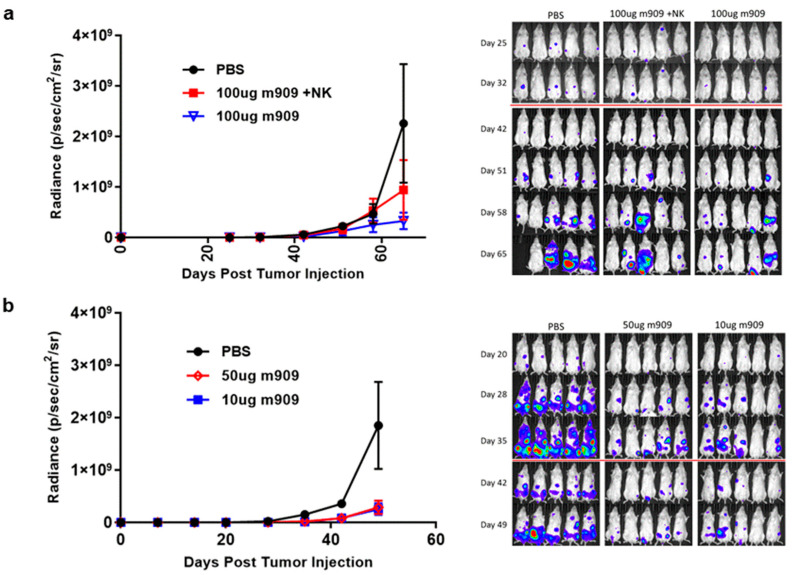
Preclinical activity of m909 against THP-1 tumors in vivo. (**a**) Mice were injected with 2 × 10^6^ THP-1 cells expressing GFP-fLuc on day 0 and imaged weekly. They were treated with IP injection starting on day 2 every 2–3 days thereafter for 10 doses with 100 μg m909 or PBS. The NK group received activated NK cells on day 2. The average maximum radiance of each group at each measurement time point is shown in the graph. Significant differences were identified on day 65 between the treatment groups and the PBS group. (**b**) Mice were injected with 2 × 10^6^ THP-1 cells expressing GFP-fLuc on day 0 and received antibody via IP injection starting on day 2 and every 2–3 days thereafter at the respective doses for a total of 10 doses. Significant differences were identified between the treatment groups and the PBS group on day 49. All error bars represent standard error. Red lines on images indicate a change in exposure time.

Our in vitro results clearly indicate that ADCC appears to be the most efficient mechanism by which m909 causes cytotoxicity in cell lines as well as against primary AML cells expressing FRβ. Therefore, we tested the cytotoxic activity of m909 in vivo using an NOD/SCID/γchain−/− (NSG) xenograft model of AML in combination with NK cell transfer. Each mouse received an intravenous (IV) injection of two million GFP-fLuc expressing THP-1 cells, followed two days later by a single IV injection of two million activated NK cells or phosphate-buffered saline (PBS) in combination with intraperitoneal (IP) injection of 100 μg of m909 or PBS. IP injection of m909 was repeated every 2–3 days for 10 doses and tumor growth was longitudinally monitored using bioluminescence imaging. A statistically significant decrease in tumor progression was observed on day 65 in the 100 μg m909 plus NK cell group, compared to the PBS group, using two-way ANOVA (Figure 4a). Notably, the 100 μg m909 alone group also had significantly lower tumor volume compared to the PBS group, indicating that the m909 antibody activity in the xenograft model was NK cell-independent. The mean difference in tumor bioluminescence between the PBS group and both the 100 μg m909 group and 100 μg m909 plus NK group was significant, with a mean difference of 2.59 × 10^9^ (95% CI 1.61 × 10^9^–3.57 × 10^9^, *p* < 0.0001) and 2.05 × 10^9^ (95% CI 1.12 × 10^9^–2.99 × 10^9^, *p* < 0.0001) respectively. There was no significant difference between the two treatment groups. Thus, since NSG mice lack T cells, B cells, and NK cells, these results indicate that NK cells were not necessary for the anti-tumor effect of m909 in vivo. (Figure 4a).

The efficacy of m909 against AML in vivo was next tested at decreased doses of m909 in the absence of NK cells. GFP-fLuc expressing THP-1 cells were injected IV at day 0. m909 was then injected at 10 μg or 50 μg on day 2 and thereafter every 2–3 days for a total of 10 doses. Tumor progression was monitored by bioluminescence imaging. Both doses of m909 showed a statistically significant reduction in tumor growth compared to the control group on day 49 (Figure 4b). The mean difference in bioluminescence between the PBS group and the 50 μg m909 group was 1.25 × 10^9^ (95% CI 6.65 × 10^8^–1.84 × 10^9^, *p* < 0.001) and between the PBS group and the 10 μg m909 group was 1.29 × 10^9^ (95% CI 6.99 × 10^8^–1.87 × 10^9^, *p* < 0.001). There was no significant difference between the 10 μg and 50 μg doses by day 49 (*p* = 0.989). Additionally, a similar experiment was conducted to compare treatment with PBS, 50 μg m909, and 100 μg of a non-specific antibody Herceptin. There was a significant tumor reduction in the mice treated with 50 μg m909 compared to PBS. In contrast, no difference in tumor growth was observed between the mice that received Herceptin and those that received PBS (Supplemental Figure S1).

### 2.5. Mechanisms for In Vivo Reduction of AML Tumor Growth

**Figure 5:**
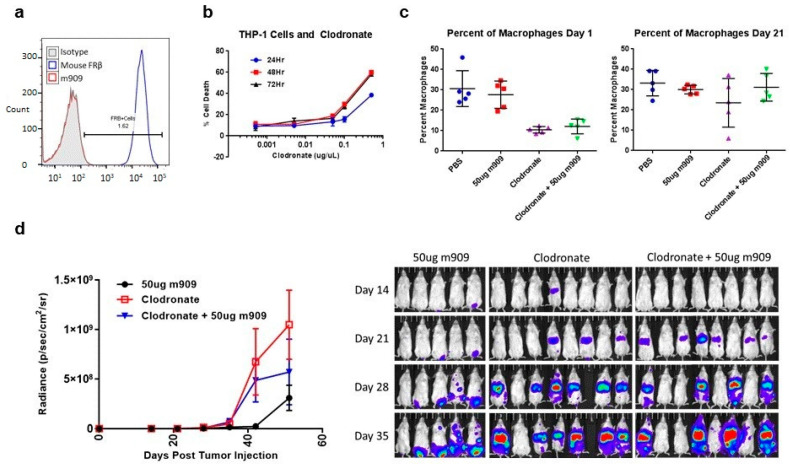
m909 activity in vivo after macrophage depletion. (**a**) m909 does not cross react with mouse FRβ. (**b**) In vitro co-culture of THP-1 cells in the presence of liposomal clodronate at different concentrations for 24, 48, and 72 h shows increasing toxicity at higher concentrations. Error bars represent standard deviation. (**c**) Blood draws from five mice in each treatment group demonstrate the percentage of macrophages on day 1 and day 21. 300 μL of liposomal clodronate was given on day −1 and repeated small dose of 25μL every 5 days until the end of antibody dosing. Error bars represent standard deviation. (**d**) Mice injected with 2 × 10^6^ THP-1 cells on day 0 and then treated with m909 for 10 doses starting day 2 as in previous experiments as well as liposomal clodronate. m909 still has an impact on tumor growth even in the presence of liposomal clodronate. Error bars represent standard error.

The previous experiments demonstrated that the addition of NK cells did not affect the ability of m909 to impact tumor growth and that even lower doses of m909 were effective against tumor growth. Therefore, we conducted studies to identify additional mechanistic pathways that could potentially be responsible for the suppression of tumor outgrowth in vivo. To determine whether m909, the anti-human FRβ specific antibody, could be binding to mouse cells, experiments were run to assess cross-reactivity. ID8 mouse cells previously engineered to express mouse FRβ (mFRβ) by lentiviral transduction were stained using anti-mouse FRβ (CL10) [31] or the anti-human FRβ (m909) and analyzed by flow cytometry. The CL10 antibody stained ID8-FRβ cells, while m909 did not, confirming a lack of cross-reactivity (Figure 5a). Our in vitro assays using human cells indicate that m909 itself does not appear to reduce cell growth, induce apoptosis, or initiate complement-dependent cytotoxicity (CDC) at a significant level. In addition, CDC is not likely to function in vivo as NSG mice lack C5 complement and therefore the ability to create the membrane attack complexes necessary for cytotoxicity through the complement cascade [32].

We next investigated whether another innate element in the NSG mice affects antibody function. While NSG mice lack NK cells, T cells, or B cells, they do harbor macrophages, albeit with impaired function [33]. We, therefore, investigated whether these residual mouse macrophages play a role in m909 activity in vivo. The drug liposomal clodronate depletes macrophages in in vivo mouse models [32,33,34,35,36,37]. Prior to introducing liposomal clodronate to mice, we evaluated the impact of liposomal clodronate on the THP-1 tumor cells in vitro. THP-1 cells were co-cultured with various doses of liposomal clodronate for 24, 48, and 72 h and then analyzed for cell death in a luciferase assay (Figure 5b). As the concentration of liposomal clodronate increased above 0.1 μg/μL, there was a significant increase in cell death. At 0.5 μg/μL, 38.5% of the cells were dead after 24 h. This increased to 59.9% at 48 h and sustained at 58.1% at 72 h. Nevertheless, this in vitro study may not reflect the function of liposomal clodronate in vivo because clodronate released from dead macrophages or residing in the culture medium cannot diffuse from the medium whereas in vivo it has a very short half-life as it is rapidly cleared by the kidneys [30,31].

We used liposomal clodronate as an agent of macrophage depletion in NSG mice and treated them with 50 μg m909, 50 μg m909 plus liposomal clodronate, or liposomal clodronate alone to control for the impact that liposomal clodronate might have on THP-1 cells. Dosing of liposomal clodronate was determined from preliminary trials as well as a literature search [35,36,37,38,39,40]. Mice were treated with 200 μL IV and 100 μL IP of liposomal clodronate one day (day −1) prior to IV injection of 2 million THP-1 tumor cells (day 0) and then treated with 25 μL doses every 5 days for the duration of antibody injections. Blood draws were performed on five mice from each group on day 1 (prior to m909 administration) and the blood was stained for macrophage markers CD11b+ and F4-80. In the absence of liposomal clodronate treatment, mice had an average of 30.6 ± 8.8% macrophages in the PBS control group and 27.7 ± 6.7% in the 50 μg m909 treated group. A lower frequency of macrophages was detected in the liposomal clodronate groups with an average of 10.4 ± 1.6% in the liposomal clodronate alone group, and 12.1 ± 3.6% in the liposomal clodronate plus 50 μg m909 group. This resulted in a statistically significant decrease in the percentage of macrophages in the groups that received liposomal clodronate, compared to those that did not. At this time point (day 1), none of the groups had yet received the antibody, therefore no experimental difference existed in the groups that went on to receive m909 from those that did not. This assay was repeated on day 21 to detect macrophage levels and the difference in the percentage of macrophages was no longer statistically significant. The PBS group had 33.2 ± 6.3% macrophages and the 50 μg m909, liposomal clodronate, and liposomal clodronate plus 50 μg m909 groups had 30.0 ± 2.1%, 23.5 ± 12.0%, and 31.2 ± 6.8% respectively (Figure 5c), indicating that the 25 μL booster doses given to the mice every 5 days did not sustain the suppression of the macrophage population.

The mice also underwent bioluminescent imaging to analyze tumor growth. The tumors in the groups that received liposomal clodronate grew more than in the 50 μg m909 alone group indicating that THP-1 cell viability was not inhibited by liposomal clodronate, in contrast to the in vitro findings. Of note, the mean tumor bioluminescence in the group that received liposomal clodronate plus 50 μg m909 was higher than the 50 μg m909 alone group at days 35, 42, and 51. This trended toward a significant difference between the 50 μg m909 group and the liposomal clodronate plus 50 μg m909, particularly at day 42 with a mean difference of 5.27 × 10^8^ (*p* = 0.069). However, this significance was not maintained at day 51 (*p* = 0.483) (Figure 5d). The data were compared using two-way ANOVA analysis.

There was also a statistically significant increase in tumor growth in the liposomal clodronate alone group, compared to the 50 μg m909 alone group at days 35, 42, and 51. The mean difference in tumor bioluminescence was 6.49 × 10^8^ (*p* = 0.009) on day 42 and 7.38 × 10^8^ (*p* = 0.003) on day 51. The addition of 50 μg m909 to the mice treated with liposomal clodronate showed a trend toward improvement which was, however, not statistically significant. Interestingly, almost all mice in the liposomal clodronate group developed tumors while the small tumors observed on day 21 in the clodronate plus 50 μg m909 group decreased, demonstrating the potential efficacy of the antibody correlating with the return of the macrophage population (Figure 5d). There was one mouse in each of the groups that did not appear to grow any tumor. The exact role that mouse macrophages are playing in the efficacy of the m909 antibody remains uncertain but there is clearly additional tumor growth when macrophages are depleted.

### 2.6. Tumor-Associated Macrophages in Ovarian Ascites Samples Express FRβ

**Figure 6:**
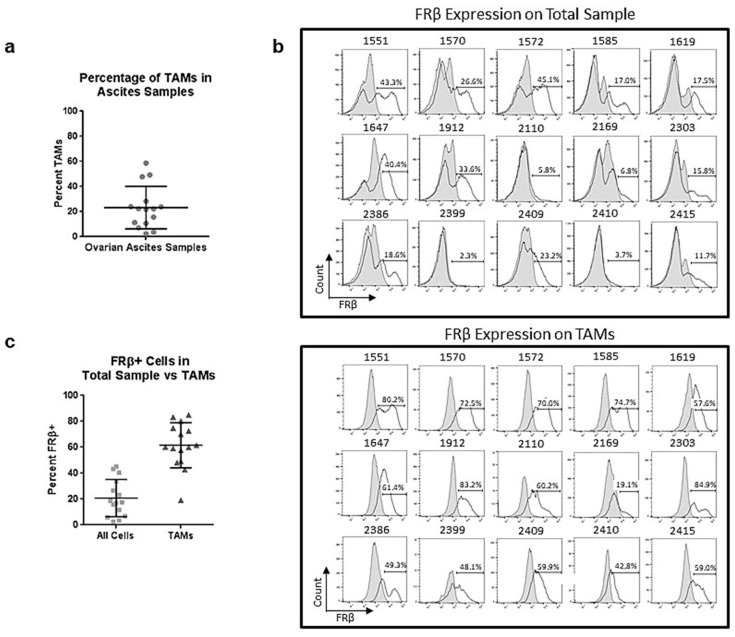
Tumor associated macrophages in ovarian cancer ascites samples and expression of FRβ. (**a**) Percentage of TAMs making up each of the fifteen ovarian ascites samples as determined by CD11b and CD14 double-positive population with an average of 23.3%. (**b**) Flow plots showing the expression of FRβ in each of the 15 total ascites samples compared to expression on TAMs in the sample. (**c**) Comparison of the average expression of FRβ in the total sample (20.8%) versus expression on TAMs (61.6%).

Having established that m909 can cause ADCC in vitro against primary AML cells and in vivo in a humanized mouse model, we also examined the application against the tumor microenvironment targeting tumor-associated macrophages (TAMs) in solid tumors using ovarian cancer. We analyzed 15 individual primary ovarian ascites samples for the percentage of TAMs in each sample as well as for the expression of FRβ in the total sample and then specifically on TAMs by flow cytometry. TAMs were defined by the CD14 and CD11b double-positive population in the samples. Overall, the ascites samples contained an average of 23.3% TAMs with a range of 2.3–58.6%. (Figure 6a). In the total samples, FRβ expression was found on an average of 20.8% of the cells with a range of 3.7–45.1%. When samples were gated for TAMs, the average expression of FRβ was 61.6% with a range of 19.1–84.9% (Figure 6b). The majority of the FRβ expression in these samples was noted to be on the TAMs.

### 2.7. m909 Can Cause ADCC of TAMs in Primary Ascites Samples

**Figure 7:**
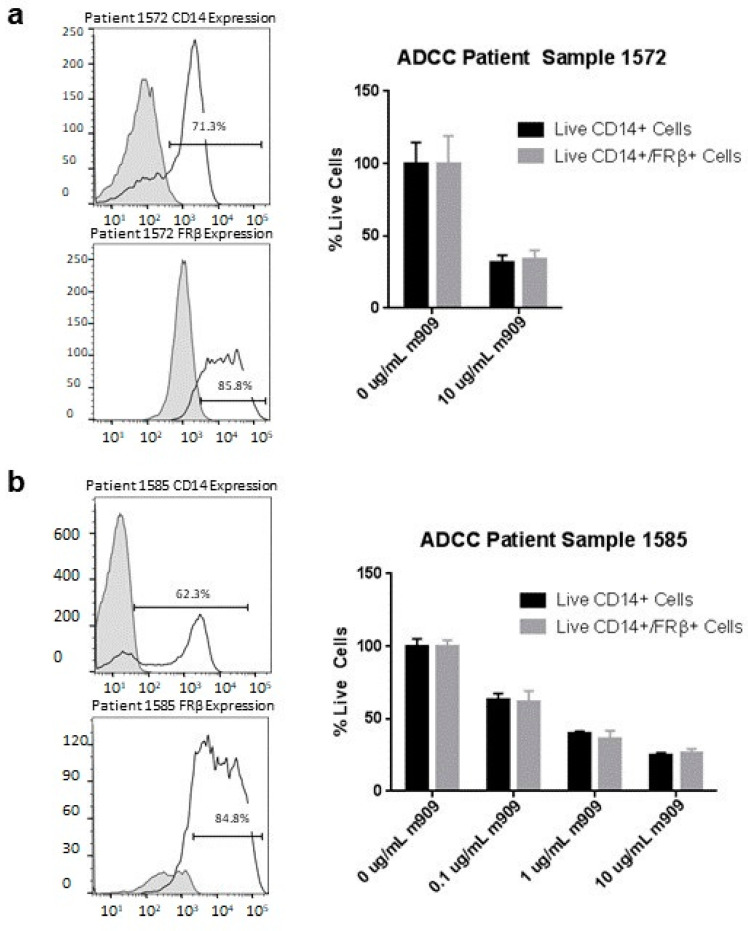
Expression of FRβ and ADCC on primary ovarian ascites samples. (**a**) CD14 and CD14/FRβ expression data for patient sample 1572. ADCC data for patient sample 1572 showing a significant decrease in live FRβ expressing cells. (**b**) CD14 and CD14/FRβ expression on patient sample 1585. ADCC data for patient sample 1585 showing a dose-dependent response to m909 administration. All samples were tested in triplicate and all graphs were normalized to wells with 0 μg/mL of m909. Error bars represent standard deviation.

Next, we explored the ability of m909 to cause ADCC of TAMs in primary ascites samples. Two ovarian ascites samples were selected that contained a high level of TAMs, which were sorted from samples using CD11b magnetic beads to isolate the positive population. ADCC by flow was then performed using a similar technique as for AML samples. Patient sample 1572 had 85.8% expression of FRβ on TAMs and when ADCC assay was performed using m909 at 10 μg/mL this resulted in a 66.1% (95% CI 36.4–95.7%, *p* < 0.001) decrease in the number of live FRβ positive TAMs (Figure 7a). We then expanded the concentration ranges to analyze activity against sample 1585, which had an FRβ expression of 84.8%. There was a clear dose-dependent response with a significant decrease compared to control at all antibody concentrations. There was a decrease in the percentage of live TAMs of 38.5% (95% CI 27.8–49.3%, *p* < 0.001), 64.3% (95% CI 53.6–75.0%, *p* < 0.001), and 74.1% (95% CI 63.4–84.9%, *p* < 0.001) at 0.1 μg/mL, 1 μg/mL, and 10 μg/mL m909 respectively demonstrating the potential of m909 to reduce TAMs in the tumor microenvironment of ovarian cancer (Figure 7b).

## 3. Discussion

Acute myeloid leukemia and ovarian cancer are both deadly diseases with a poor overall prognosis. AML is one of the most common types of leukemia with a five-year survival of only 25% [41]. Initial treatment of AML with induction chemotherapy has not changed significantly in the past 30 years and 60–85% of those under age 60 will achieve complete response while only 40–60% of older patients will [42]. Following remission, patients receive consolidation chemotherapy and/or hematopoietic cell transplant that achieves cure in 60–70% of those with favorable risk factors. However, those with intermediate or poor risk profiles only have a 10–15% chance of cure so new treatments are needed to improve these outcomes [42]. Similarly, ovarian cancer is the deadliest gynecologic cancer and there are no good screening tools, resulting in patients typically being diagnosed at stage III or IV disease where the five-year survival is 29% [41]. The majority of patients will respond to initial chemotherapy. However, like AML, approximately 80% will recur, making the development of new treatment strategies imperative.

In this study, we validated m909, an anti-human FRβ antibody that demonstrates in vitro efficacy via ADCC against AML cells in a dose- and expression-dependent manner for both established cell lines and primary acute myeloid leukemia cells. m909 does not appear to cause significant cytotoxicity in vitro by any of the other mechanisms analyzed including CDC, apoptosis, and growth inhibition. We further explored the efficacy of m909 in a mouse model of AML and found that tumor growth is significantly reduced by m909. The exact mechanism by which m909 functions to inhibit tumor growth in vivo is still unclear. Although NK cells were required in vitro for efficacy, interestingly, administration of m909 to tumor-bearing NSG mice had similar efficacy in reducing the tumor growth over time with and without the infusion of NK cells. NSG mice lack the majority of immune cell elements including T cells, B cells, NK cells, and complements which make them an excellent resource for the development of humanized models. However, macrophages and dendritic cell populations are still present in the NSG model, although the NOD genetic background does result in a reduction in function for these populations in vivo [43]. This suggests a possible role of innate effector cells, such as mouse macrophages, as mediators of ADCC in our in vivo model.

Our results demonstrate that there is no cross reactivity between the anti-human FRβ antibody and mouse FRβ since m909 did not bind to mouse ID8 cells with engineered expression of mouse FRβ. This would also indicate that mouse macrophages are unlikely to interact with m909 via the human FRβ-binding portion of the antibody. However, reactivity between the Fc region of m909 and the mouse macrophage cells may result in antibody-dependent cellular phagocytosis, as some human IgG antibodies can react with mouse Fcγ receptors [44]. Despite the fact that NSG mice harbor mutations leading to defective macrophages, other studies have shown these macrophages can impact model systems using human hematopoietic cells and have validated the use of liposomal clodronate to deplete these macrophages [35,36,37,38,39,40,43].

We hypothesized that macrophages were involved in the m909 mediated response in our in vivo system and used liposomal clodronate for depletion. Our results demonstrated that mice receiving the liposomal clodronate with m909 trended toward larger tumors compared to those receiving m909 alone, possibly indicating some impact on antibody function, however, the difference was not statistically significant. Interestingly, some mice receiving m909 and liposomal clodronate had tumors that were detectable on day 21 but disappeared on day 35, suggesting the efficacy of m909 in these mice. This finding correlates with our data demonstrating an initial reduction in macrophages on day 1 but failing to achieve persistent depletion with continued small doses of liposomal clodronate by day 21. If indeed mouse macrophages are playing a role in facilitating cytotoxicity of the tumors with m909, the normalization by day 21 may be responsible for the disappearance of these tumors. Perhaps treatment with higher dosing of the liposomal clodronate would allow for sustained macrophage depletion response yielding more informative in vivo data, however, this must be balanced with the established toxicity that NSG mice experience with liposomal clodronate dosing [35]. While the mechanism of action of m909 in the in vivo model is not entirely resolved, what is evident is that there is a significant impact of the antibody on reducing tumor growth in both in vitro and in vivo models, rationalizing the further development of this therapeutic approach.

In addition to direct therapy against AML, our results also demonstrate expression of FRβ on TAMs from 15 individual ovarian ascites samples as well as the ability for m909 to cause ADCC of TAMs in a dose-dependent manner. Elimination of M2 polarized macrophages from the tumor microenvironment with m909 could potentially improve the efficacy of chemotherapy or immunotherapy treatments targeting the tumor cells directly when used in combination. M2 polarized TAMs are also not unique to ovarian cancer and occur in the microenvironment of many solid cancers making their application broader [21,45]. Unfortunately, there are also challenges in replicating the tumor microenvironment and further experiments to demonstrate the efficacy of m909 in vivo or in combination with other therapeutic agents requires transitioning away from a human NK reconstituted mouse model, as a functional immune system is necessary to represent the tumor microenvironment.

Research in immunotherapies for various cancers has led to breakthrough treatments in some diseases with durable responses unlike any previously seen with standard chemotherapy, radiation, or surgery [46]. Our data demonstrate FRβ as a potential therapeutic target for both direct cancer therapy causing cytotoxicity to AML cells in both in vivo and in vitro models, as well as indirect therapy targeting the tumor microenvironment of solid tumors such as ovarian cancer through in vitro cytotoxicity of TAMs. The mechanism of action in the in vivo AML model remains unclear, but our data suggest that mouse macrophages may play at least a partial mechanistic role in m909 activity. Further investigation will be necessary to clarify the in vivo mechanisms of m909 activity in order to better understand how m909 might be used in the treatment of AML. Additionally, studies aimed at better understanding its application in targeting the tumor microenvironment and its potential as a dual therapeutic in the treatment of solid tumors warrant consideration.

## 4. Materials and Methods

### 4.1. Antibodies

m909 FRβ directed antibody was described earlier [27], and provided by Monojul for all in vitro and in vivo studies. Anti-CD33_PE (cat# 366607), anti-CD14_PE-Cy7 (cat# 367111), anti-CD34_FITC (cat# 343603), anti-F4-80_PE-Cy7 (cat# 123113), anti-CD11b_PE (cat# 101207), and anti-mouse FRβ_APC (cat# 153305) antibodies were ordered through Biolegend.

### 4.2. Cell Lines

All cell lines were cultured in complete media (RPMI-1640-GlutaMAX with 10% fetal bovine serum, 100 U/mL penicillin, and 100 μg/mL streptomycin) at 37°C. THP-1 and MV4-11 cells were purchased from American Type Culture Collection and HL-60 cells were kindly provided by Gwenn Danet-Desnoyers (University of Pennsylvania). CHO-FRβ cells were a gift from the lab of Phillip Low at Purdue University and CHO-K1 cells were purchased from American Type Culture Collection. All cell lines were transduced with a lentiviral vector containing GFP and fLuc separated by a T2A ribosomal skipping element (GFP-2A-fLuc). De-identified natural killer cells and peripheral blood mononuclear cells collected from healthy donors were purchased from the University of Pennsylvania Human Immunology Core (HIC). George Coukos kindly provided the ID8 cell line. The parental ID8 line was transduced with a lentiviral construct containing mCherry (RFP) and fLuc (RFP-2A-fLuc) to create ID8-RFPfLuc. ID8-RFPfLuc cell line was then transduced with a lentiviral construct encoding murine FRβ cDNA (Origene) to produce ID8.mFRβ-RFPfLuc.

### 4.3. Flow Cytometry

m909 and human IgG1 antibody were biotinylated using the EX-Link Sulfo-NHS-LC Biotinylation Kit (Thermo Fisher Scientific). Up to 1 × 10^6^ cells were labeled in 100 μL staining buffer (2% fetal bovine serum in phosphate-buffered saline) containing relevant antibodies at 4 °C. Streptavidin(SA)-allophycocyanin(APC) was used for secondary detection of FRβ. Samples were also stained with other antibodies as indicated in each study as well as with the LIVE/DEAD ™ Fixable Aqua Dead cell stain. Samples were assessed by flow cytometry using a BD LSRFortessa Flow Cytometer, and results were analyzed with FlowJo 10.4.2 software.

### 4.4. Antibody-Dependent Cellular Cytotoxicity (ADCC) in Cell Lines

THP-1, MV4-11, HL-60, CHO-FRβ, and CHO-K1 cells transduced with GFP-2A-fLUC lentiviral vector were used in ADCC assays. 1 × 10^4^ cells were plated in triplicate in a 96 well plate in 100 μL phenol-free complete media. Adherent cell lines were plated the day prior to the experiment to allow for adherence and fresh media exchanged on the day of the experiment. m909 was added at concentrations indicated in each study and incubated with cells for 20 min. NK cells were then added at an E:T ratio of 10:1 and co-cultured overnight. Residual luciferase activity was then measured using the Extended-Glow Luciferase Reporter Assay System (Life Technologies, Carlsbad, CA, USA). Percent of lysis was calculated as follows: (100 − ((average signal antibody-treated wells)/(average signal cells alone) × 100)).

### 4.5. Cell Growth Assays

THP-1, MV4-11, and HL-60 cells were plated at a concentration of 0.3 × 10^6^ cells/mL in six well plates with and without m909, and the growth was calculated by measuring cell counts daily. The same experiment was performed for CHO-FRβ and CHO-K1 cells except four wells with 0.5 × 10^5^ cells/well were plated in 24 well plates for each cell line and one well was counted daily. All experiments were performed in triplicate.

### 4.6. CDC Assays

THP-1, HL-60, CHO-FRβ, and CHO-K1 cells transduced with GFP-2A-fLUC were plated in flat-bottom 96 well plates (10,000 cells/well) with different concentrations of antibody in media containing human serum. After 3 h incubation at 37 °C in a 5% CO2 incubator, samples were analyzed for residual luciferase activity using the Extended-Glow Luciferase Reporter Assay System (Thermo Fisher Scientific #T1034) as with the ADCC assays, and percent cell lysis was calculated in the same fashion

### 4.7. Apoptosis Assays

THP-1, HL-60, CHO-FRβ, MV4-11, and CHO-K1 cells (0.3 × 10^6^ cells/well) in a 12 well plate were incubated in media containing 10 μg/mL of m909 for 24 h. Samples were then stained for apoptosis markers (PE Annexin V, Biolegend #640908 and 7-AAD, Biolegend #420404) to detect loss of plasma membrane integrity and analyzed for percentage of cells in early and late apoptosis using BD LSRFortessa flow cytometry.

### 4.8. Antibody-Dependent Cellular Cytotoxicity in AML Patient Samples

De-identified primary AML patient samples were purchased from the Tissue Bank & Cell Processing facility in the Stem Cell & Xenograft Core, University of Pennsylvania. Samples were stained with fluorophore-labeled antibodies for their expression of FRβ, CD34, CD33, and CD14 by flow cytometry. A sample with a higher expression of CD33 and FRβ was selected for ADCC. Samples were thawed and 3 × 10^4^ cells were plated in a 96 well plate in triplicate and m909 was added at the specified concentrations for 20 min. NK cells purchased from the HIC core were then added at an E:T ratio of 10:1. Following 24 h incubation, the cells were stained for CD33, FRβ, and LIVE/DEAD ™ Fixable Aqua. Brightcount counting beads were added and the total numbers of CD33+ live cells and CD33+/FRB+ live cells were calculated at the different antibody concentrations using flow cytometry. This technique was also performed using THP-1 cells for proof of concept.

### 4.9. Antibody-Dependent Cellular Cytotoxicity in Ovarian Cancer Patient Ascites Samples

De-identified primary ovarian cancer ascites samples were provided by the University of Pennsylvania Tumor Tissue and Biospecimen Bank. Samples were analyzed by flow cytometry for expression of FRβ, CD14, and CD11b. Patient samples used for ADCC were then sorted by CD11b expression to isolate macrophages using CD11b magnetic beads. Using the same technique as the AML samples, 3 × 10^4^ cells from the CD11b positive population were plated per well in a 96 well plate in triplicate, and m909 was added at the specified concentrations for 20 min. NK cells purchased from the HIC core were then added at an E:T ratio of 10:1. Following 24 h incubation, the cells were stained for CD14, FRβ, and LIVE/DEAD ™ Fixable Aqua. Brightcount counting beads were added and the total numbers of CD14+ live cells and CD14+/FRβ+ live cells were calculated at the different antibody concentrations using flow cytometry.

### 4.10. Mice

Female NOD/SCID/γchain−/− (NSG) mice that were 8 to 12 weeks old were purchased from the Stem Cell Xenograft Core. They were treated and maintained under strict pathogen-free conditions. All protocols were approved by the University of Pennsylvania Institutional Animal Care and Use Committee. THP-1 cells transduced with lentiviral GFP-2A-fLuc vector were sorted by flow cytometry to obtain a 100% GFP expressing population. Cells were analyzed by flow cytometry for FRβ expression and then 2 × 10^6^ cells were injected intravenously (IV) into mice on day 0. Five mice were included in each treatment group for all except for studies in which liposomal clodronate was used. These studies included 10 mice/group, due to immediate death of some mice following IV liposomal clodronate injection in a pilot study, possibly due to injection toxicity. In studies including NK cells, NK cells were purchased from the HIC, activated with IL-2, and 2 × 10^6^ cells were injected IV into the tail vein on day 2 following tumor inoculation. Antibodies were administered via intraperitoneal injection (IP) every Monday, Wednesday, and Friday for 10 doses starting on day 2 following tumor injection. For studies requiring macrophage depletion, mice were treated with liposomal clodronate. Liposomal clodronate (5 mg/mL) was purchased from Liposoma B.V. (Amsterdam, The Netherlands) and 200 μL were given IV and 100 μL IP on day −2 and then 25 μL IV on day 1 and every 5 days until the end of antibody administration. Dosages were extrapolated from a review of the literature using liposomal clodronate in mice [30,31,32,33,34,35,36,37] and from pilot study experiments.

Mice were euthanized if they lost at least 20% of their weight, if they developed ascites or if they exhibited any sign of suffering that could not be relieved.

### 4.11. Bioluminescence Imaging

Bioluminescence imaging studies were performed using the Lumina IVIS imaging system and quantified with the Living Image Software (PerkinElmer). Mice were injected intraperitoneally with D-luciferin (150 mg/kg) and imaged under isoflurane anesthesia. Images were recorded until two consecutive images showed decreasing signal and peak signal was determined for each mouse at each designated time point. Pseudocolor images representing light intensity were generated with Living Image Software.

## Data Availability

There are no large datasets used in this manuscript and all raw data is available upon request.

## Supplementary Materials

The following are available online at https://www.mdpi.com/article/10.3390/ijms22115572/s1, Supplemental Figure S1 Preclinical activity of m909 against THP-1 tumors compared to non-specific antibody in vivo. Mice were injected with 2 × 106 THP-1 cells expressing GFP-fLuc on day 0 and imaged weekly. They were treated with IP injection starting on day 2 every 2–3 days thereafter for 10 doses with 50 μg m909, 100 μg Herceptin, or PBS. The graph demonstrates the average radiance of each group at each measurement time point. Error bars represent standard error. A significant difference in average maximum radiance of 1.226 × 10^9^ (95% CI 1.007 × 10^7^–2.441 × 10^9^) was identified on day 49 between the 50 μg m909 group and the PBS group. There was no difference between the 100 μg Herceptin and PBS group.
