# Supplementary material for: Folate Receptor Beta as a Direct and Indirect Target for Antibody-Based Cancer Immunotherapy
Source: Int J Mol Sci. 2021 May 25;22(11):5572. doi: 10.3390/ijms22115572 (PMC8197521; doi:10.3390/ijms22115572)
Supplement: Supplementary file 1 [file ijms-22-05572-s001.zip › Supplementary/ijms-1134568-final-suppl.pdf]

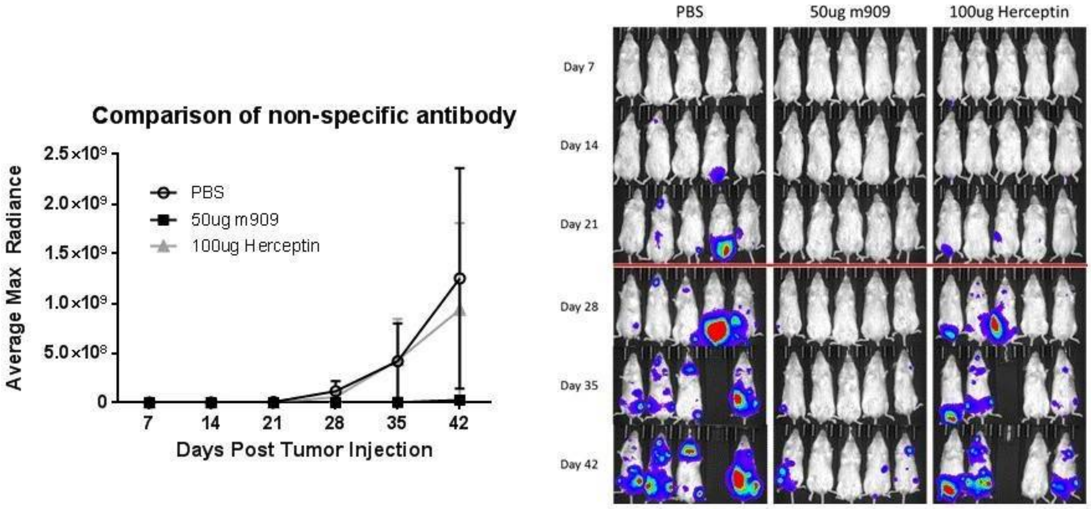

Supplemental Figure S1 Preclinical activity of m909 against THP-1 tumors compared to non-specific antibody *in vivo*. Mice were injected with 2×10^6 THP-1 cells expressing GFP-fLuc on day 0 and imaged weekly. They were treated with IP injection starting on day 2 every 2–3 days thereafter for 10 doses with 50ug m909, 100ug Herceptin, or PBS. The graph demonstrates the average radiance of each group at each measurement time point. Error bars represent standard error. A significant difference in average maximum radiance of 1.226×10^9 (95% CI 1.007×10^7-2.441×10^9) was identified on day 49 between the 50ug m909 group and the PBS group. There was no difference between the 100ug Herceptin and PBS group.
